# Patient-Specific Implants for Pelvic Tumor Resections

**DOI:** 10.3390/jpm11080683

**Published:** 2021-07-21

**Authors:** Kevin Döring, Kevin Staats, Stephan Puchner, Reinhard Windhager

**Affiliations:** Division of Orthopaedics, Department of Orthopaedics and Trauma Surgery, Medical University of Vienna, 1090 Vienna, Austria; kevin.doering@meduniwien.ac.at (K.D.); kevin.staats@meduniwien.ac.at (K.S.); stephan.puchner@meduniwien.ac.at (S.P.)

**Keywords:** pelvic tumors, 3D printed prostheses, computer aided design pelvic reconstruction, arthroplasty, complications, bone tumor, pelvis

## Abstract

Introduction Limb salvage surgery for periacetabular malignancies is technically demanding and associated with a considerable likelihood of postoperative complications and surgical revision. Reconstruction using custom-made implants represents the treatment of choice. This study was conducted to analyze treatment outcomes of custom-made implants in a single orthopaedic tumor center. Patients and Methods Twenty patients with a histologically verified periacetabular malignancy and a median follow up time of 5 (1–17) years were included. Results The median number of revision surgeries per patient was 1.5 (0–7). Complications were dislocations in 3 patients, aseptic loosening in 4 patients, deep infections in 9 patients, thromboembolic events in 5 patients and sciatic nerve lesions in 4 patients. Overall survival was 77% after one year, 69% after two years and 46% after five years. Median Harris Hip Score was 81 (37–92) points at last follow up. Conclusion Although internal hemipelvectomy and reconstruction using custom-made implants is linked with a high risk of postoperative complications, good functional outcomes can be regularly achieved. This information may help treating surgeons to find adequate indications, as eligible patients need to be critically selected and integrated into the decision-making process.

## 1. Introduction

Limb sparing surgery of primary malignant pelvic tumors has become the treatment of choice over the last decade, mainly due to improvement in surgical technique, imaging and perioperative management [1,2]. However, limb-sparing surgery remains challenging with respect to defect reconstruction and management of complications [3]. Among the three types of resections and reconstructions described by Enneking and Dunham in 1978, involvement of the acetabulum (Type 2) remains the most challenging area, whereas Type 1, resection involving the ileum and Type 3, resection involving the pubis and ischium require less or only minimal reconstruction [4]. Several methods have been applied for reconstruction of the acetabulum, such as iliofemoral arthrodesis or pseudarthrosis, allograft reconstruction, irradiated, autoclaved or frozen autografts, femoral neck autografts and allograft-prosthetic composites, all of them being associated with a higher complication rate than simple excision arthroplasty or transposition of the hip [2,5,6,7]. Thus, low complication rates and a possible fast recovery with satisfactory functional outcomes are important factors influencing the decision-making process with patients.

Endoprosthetic replacement bears the advantage of immediate stability and allows early weight bearing, which is of utmost importance in this mainly young patient group. Among the endoprosthetic replacement, custom-made endoprostheses have been used even in the last three decades and still represent the technique of choice due to high variability in pelvic anatomy [8]. In cases when part of the iliac crest can be spared, other implants like saddle prostheses or ice-cream cone endoprostheses have been applied [9,10]. These endoprostheses offer the advantage of immediate availability but come with the drawback of limited adaptability during the procedure. Three-dimensional (3D) printing has revolutionized the production of custom-made implants. While in the beginning, 3D models have been produced with the help of CAD techniques by milling or laser printing of raisin which served as templates to produce the endoprosthesis, 3D printing of metal not only allows to speed up the manufacturing process but also enables the creation of rough surfaces at the bone interface for rapid and long-lasting osseointegration. [2] One of the big advantages of this process was the improved visualization of the complex and variable pelvic anatomy, which significantly improve the accuracy of resection margins and thus help to improve local tumor control. [11] Another significant improvement was the introduction of patient specific jigs to exactly define resection planes, which is a prerequisite for a perfect match between osteotomy and custom implant [12]. This technique of thorough planning made the procedure more straight forward and reduced surgery time. 

Nevertheless, the complication rate and especially the deep infection risk of custom-made implants is significantly higher in the pelvic region compared to reconstructions of other regions, which dampened enthusiasm about this type of reconstruction [13,14]. Furthermore, as pelvic reconstruction using custom-made implants is only rarely necessary, follow up data on this type of reconstruction are rare. Thus, we conducted this study to analyze the outcome of custom-made prostheses in a single center setting over a follow-up period of three decades. 

For this, we asked the following questions:

(1) What where complication rates and revision free survivals following reconstruction with pelvic custom-made implants?
(2) What was the oncological survival after extensive pelvic tumor resection?
(3) What were functional outcomes and physical limitations?

## 2. Materials and Methods

### 2.1. Study Design

This study was conducted as a retrospective analysis of the Vienna Bone and Soft Tissue Tumor Registry at the orthopedic department of the Medical University of Vienna analyzing patients who were treated for malign pelvic bone tumors using custom made prostheses between 1990 and 2000.

### 2.2. Patients

Between (1) 1990 and 2000, 26 patients underwent resection of (2) pelvic malign bone tumors at a single center in Austria and (3) received reconstruction using custom made pelvic prostheses; those patients were considered potentially eligible for this retrospective study. Except of oncological survival analyses, 6 of these 26 patients were excluded due to a follow up below one year and, thus, no possibility of an adequate prosthesis assessment regarding function and complications. The median (range) age at surgery was 25 (13–63) years, the median follow-up after surgery was 5 (1–17) years. A total of 9 of the 20 patients were men and 11 were women. Twelve patients received postoperative chemotherapy. The median tumor size was 343 (22–3600) cm^3^ (Table 1). Because patients excluded from retrospective analyses often fare worse than patients included, we wished to analyze whether patients excluded from this study differed in important aspects. For this reason, a comparison between groups was performed. In general, patients excluded from this study due to low follow up time showed worse oncological outcomes, as all patients excluded died of disease in the year after primary resection (*p* = 0.005), but no other differences were found (Table 2). Follow-up examinations were performed in our outpatient clinic by clinical joint and radiographic assessment.

### 2.3. Surgical Approach and Extend of Reconstruction

**Figure 1:**
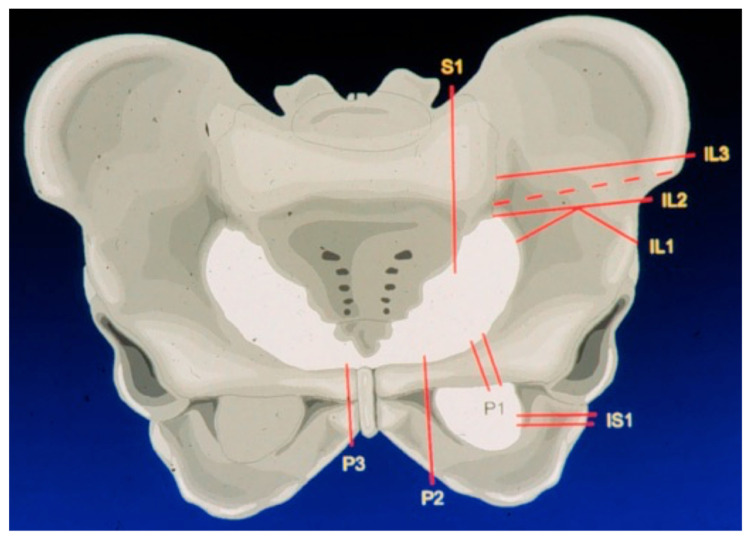
Most frequent resection lines in our cohort according to the Enneking and Dunham classification.

**Figure 2:**
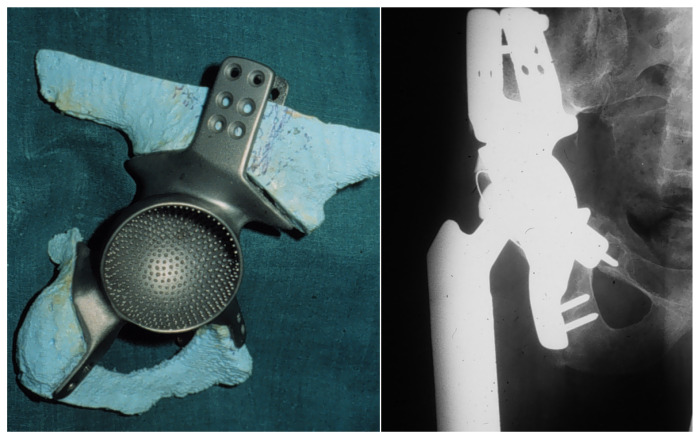
Real-size planning model for preoperative prosthesis and osteotomy planning.

Thorough preoperative planning is obligatory to attain a well-directed identification of patients eligible for an extensive resection and reconstruction linked to a potentially high level of postoperative complications. To achieve this, computer tomography (CT) and magnetic resonance imaging (MRI) were used in combination of a complete staging and assessment of relevant comorbidities. Before surgery, bowel preparation and ureter catheterization are performed. In surgery, the patient is placed in a mobile lateral position to allow for flexible intraoperative patient rearrangements. Depending on tumor extension, a ventral or combined ventral and dorsal approach, as proposed by Windhager et al., is used [11]. This type of approach allows a good intraoperative visualization of osteotomies and controlled fixation of porously coated fixation sites. Custom-made endoprostheses were provided either as single or split designs, depending on resection size and form. The extend of the pelvic resection and reconstruction was grouped according to the Enneking and Dunham classification of internal hemipelvectomies depending on resection involvement of the iliac, pubic, or ischial bone [4] (Figure 1). Custom-made prostheses were planned using computer tomography (CT) and thereafter constructed into real size planning models, on which resection lines were defined by the surgeon (Figure 2). Thereafter, the definitive individualized prosthesis (Howmedica, Kiel, Germany) was produced and implanted. Wide resection margins were achieved in 18 patients, while one patient had marginal resection. One resection was histologically deemed intralesional. Patients typically received a hip to leg plaster cast in the surgical theater until 6 weeks after surgery. Afterwards, patients were mobilized under guidance of a hip brace and subsequent weight bearing increase for another 6 weeks. Orthoses were removed 4 to 6 months after surgery.

### 2.4. Primary and Secondary Study Objectives

This studies’ primary goal was to assess prosthesis survival and postoperative complications after implantation of pelvic custom-made prostheses. To achieve this, surgical protocols, outpatient visits and discharge letters were screened for complications and revisions. Complications following custom made prosthesis implantation were grouped according to the ISOLS classification of endoprosthetic failure by Henderson et al. into type I or soft-tissue failure and dislocation, type II or aseptic loosening, type III or structural failure with periprosthetic fractures or implant breaking, type IV or deep infection or periprosthetic joint infection (PJI), and type V or tumor progression and prosthesis contamination [15]. Our secondary study objectives were the assessment of patient survival and functional outcomes. The Harris Hip Score (HHS) was used to determine functional outcomes [16].

### 2.5. Ethical Approval

Ethical approval for this study was obtained from the Ethics commission of the Medical University of Vienna.

### 2.6. Statistical Analysis

Descriptive statistics were used to detect frequencies, medians and ranges of postoperative complications. After assessment of relevant demographic and surgery related variables, we used Kaplan Meier survival analyses and log rank testing to determine revision free and total oncological survivals. To further distinguish complications regarding different reasons for revision, we differentiated complications according to the ISOLS classification to process revision specific survival analyses. We further analyzed “prosthesis explantation”, “thromboembolic events” and “sciatic nerve lesions” as additional parameters due to high prevalence. As all patients who were excluded due to low follow up died of disease in the year after surgery, these patients were included in the oncological survival calculations. Relevant parameters, such as surgical approach, extend of resection, type of femoral stem or pelvic cup and postoperative complications, were reviewed in univariate analyses using independent T-Tests to screen for a possible impact on HHS. The statistical analysis was performed with IBM SPSS version 26 (IBM). A *p* value < 0.05 was considered significant.

## 3. Results

### 3.1. Complications Following Pelvic Reconstruction 

Patients were likely to require surgical revision after implantation of custom-made implants. At the time of last follow up, four patients had no surgical revision after prosthesis implantation, while 16 patients had at least one revision (Table 3). The median number of revision surgeries per patient was 1.5 (0–7). The first surgical revision was performed with a median of 27 (0 days–6 years) days after surgery. No surgical parameters influencing revision free survival were found (Table 4). Regarding type I complications according to the ISOLS classification by Henderson et al., we found a revision free survival of 90% after one year and 84% after two and five years. Type I complications occurred in three patients suffering from dislocation of their pelvic prosthesis, which required surgical revision after a median of 5 months (14 days–20 months) after surgery. Two of these patients received open reduction, while one patient had a femoral stem change. Dislocations did not recidivate after surgical revision. Type II complications or aseptic loosening showed a revision free survival of 95% after one year, 89% after two years and 78% after five years. Aseptic loosening occurred in four patients after a median of 38.5 (10–80) months. One of these patients required stem change to a KMFTR proximal femoral modular endoprosthesis and needed two additional revisions for aseptic loosening thereafter. Type IV complications or deep infections were the most prevalent surgical complications, with 9 out of 20 patients suffering from infections which needed surgical revision after a median of 86 days (13 days–5 years) after primary prosthesis implantation. Although most of these infections could be treated with debridement and antibiotic therapy, three patients required implant removal due to otherwise uncontrollable infections after a median of 15 months (95 days–16 years) after surgery. There were no revisions due to type III complications or periprosthetic fractures, as well as type V complications or tumor progression in this study. Four patients suffered from sciatic nerve lesions, of whom two received singular surgical neurolysis with a median of 26 (24–28) months after surgery. Thromboembolic events were frequently observed after surgery, with 5 out of 20 patients suffering from thromboses. Three of these patients required immediate revision surgery at the day of prosthesis implantation, while two patients were successfully treated conservatively.

### 3.2. Oncological Survival after Extensive Pelvic Tumor Resection and Reconstruction

**Figure 3:**
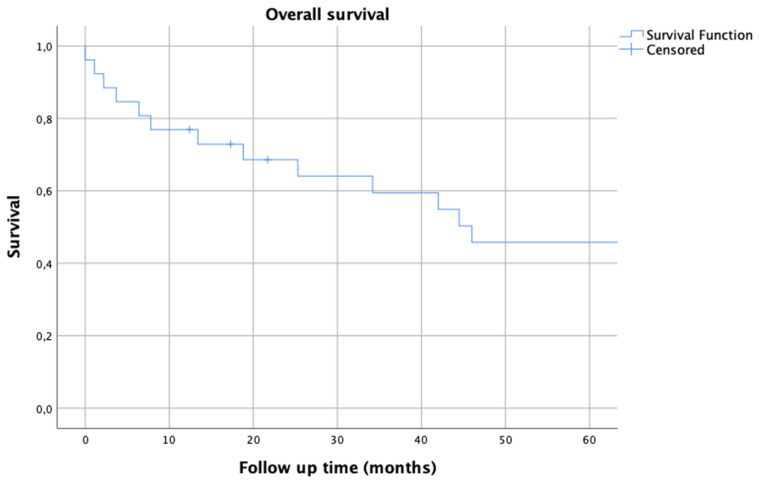
Kaplan–Meier survival analysis for overall oncological survival.

By including all patients with adequate follow up and patients with a follow up under one year due to death by disease, we found an overall survival of 77% after one year, 69% after two years and 46% after five years (Figure 3). Eight patients suffered from metastatic lesions, which occurred in the lung (*n* = 5), brain (*n* = 2), liver (*n* = 2), peritoneum (*n* = 1) and spleen (*n* = 1). Three of these patients received lobectomy, while one patient had resection of his brain metastasis.

### 3.3. Functional Outcomes and Physical Limitations

Fifteen patients with a minimum follow up of one year could be functionally assessed, while a complete Harris Hip Score could be retrieved in 11 patients, showing good results with a median score of 81 (37–92) points at time of last follow up visit at the outpatient clinic. Six patients were able to walk without walking aid and six patients needed one walking stick, while three patients were mobilized with two crutches. No information regarding walking limitations could be assessed in five patients (Table 4). We found that patients which were surgically revised for infections showed a worse HHS than patients who had no revision due to infection (59.6 versus 84.2 points, *p* = 0.033).

## 4. Discussion

**Figure 4:**
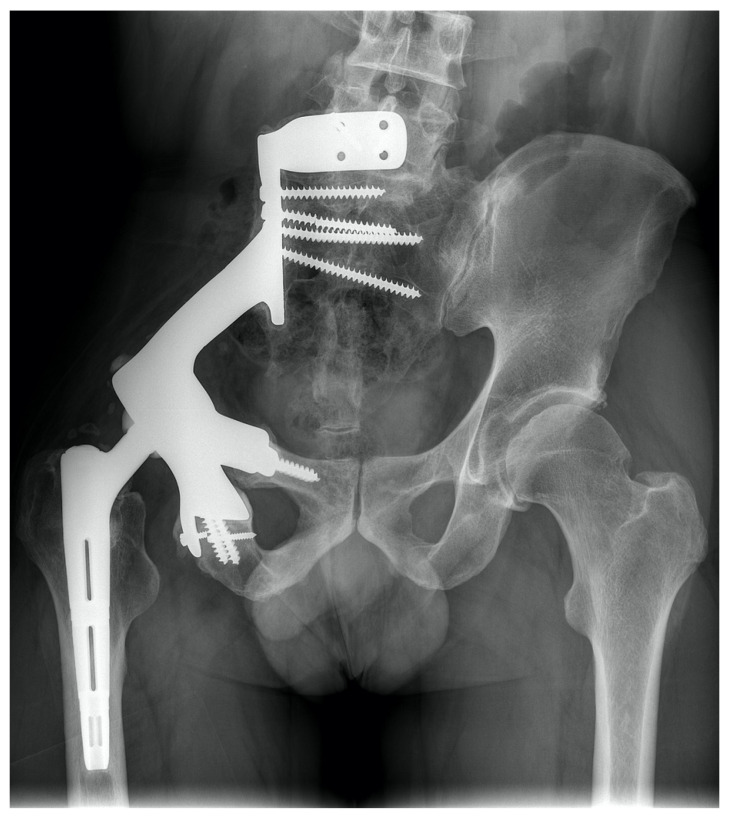
Patient 1 suffered from Ewing’s sarcoma and received wide resection via a type I and II internal hemipelvectomy through a combined ventral and dorsal approach with implantation of a 3D custom made prosthesis and articulating austroprosthesis stem in 1991. The picture shows the patient with no subjective physical limitations and walking without aid at a 19 year follow up.

Defect reconstruction using extensive custom-made pelvic implants after tumor resection is an effective but risky surgery reserved for suitable patients with large periacetabular tumors. Due to the rare indication, relatively little is known at medium- to long-term follow up. We found that postsurgical complications, such as deep infections, were very common and linked to potential prosthesis explantation with concomitant severe functional losses. We further found that patients had, in consideration of the reconstruction extend, comparatively good functional results (Figure 4). This information should promote the use of limb salvage surgery using custom-made prostheses in otherwise unsalvageable limbs and provide a scientific basis for complication and function comparisons of future custom-made prosthesis design models, such as 3D-printed custom-made prostheses.

### 4.1. Limitations

As a retrospective study, potential biases of this study type need to be disclosed.

As surgeries were performed between 1990 and 2000, not only implant types, but also surgical techniques, as well as anesthesia [17] and oncological therapies, evolved to the present date. However, indications for custom-made prostheses may still be found. Due to the lack of differentiated data, especially with regard to complications on this type of pelvic reconstruction, we nonetheless believe that this studies’ results are of importance, in particular as a potential baseline for emerging custom-made prosthesis designs and as a field report for treating surgeons.

No detailed influences on revision- or overall oncological survivals were given in statistical analyses due to low power linked to low patient numbers (*n* = 20). Although this limitation may have hampered the breakdown of more intricate findings, such as parameters related to a diminished prosthesis survival, this study aimed to give a more generalized and descriptive outlook on experiences with a rare kind of extensive pelvic reconstruction.

As only patients treated due to malignant tumors were included in this study, this studies’ results are limited and need to be considered exclusively in this patient collective. This is especially important with a recent emerge of studies describing the use of custom-made pelvic implants and components in revision arthroplasty settings, as this patient collective comes with different demographics and functional status [18,19,20].

Due to this studies’ limitation to custom-made pelvic reconstruction, no direct comparison of surgical outcomes regarding alternative reconstruction strategies can be made. As patients with periacetabular tumors receiving endoprosthetic reconstruction might differ from patients treated with biologic reconstruction, such as iliofemoral or ischiofemoral arthrodeses or coaptations, allograft reconstruction or ablation in terms of expected survival, functional outcomes and the range and frequencies of postoperative complications, results of this study should be limited to patients with presumably large and highly malignant acetabular tumors treated with custom-made endoprostheses [11].

Missing data need to be disclosed due to the potential long follow up period, as preoperative imaging was not available in every case. In cases when MRI or CT were missing, radiologic reports created by radiologic specialists were used for tumor size quantifications and localization assessments. Detailed functional scores were sometimes unavailable.

### 4.2. Complications after Extensive Pelvic Reconstructions 

**Figure 5:**
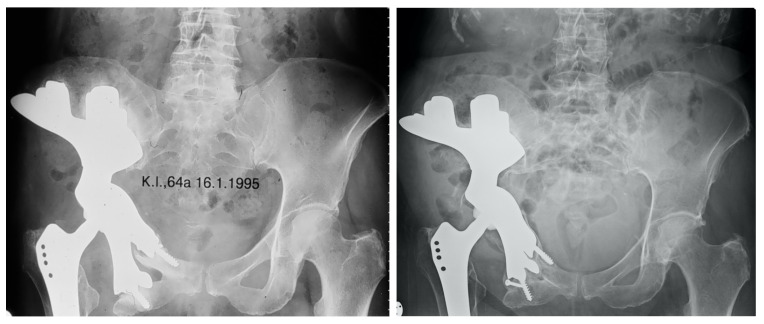
Patient 2 suffered from a periacetabular chondrosarcoma and received wide resection by a type II, III internal hemipelvectomy through a combined ventral and dorsal approach and subsequent reconstruction with a custom-made pelvic implant articulating with a Zweymueller stem in 1992. The patient suffered from aseptic loosening of the implant six years after primary surgery, which was addressed by accretion of iliac crest autograft bone. A deep vein thrombosis was treated conservatively. The X-ray, which was taken 20 years after primary surgery, shows good hip function, and the patient can walk with a walking stick and has an HHS of 89 points.

Although the use of custom-made prostheses led to primary stable reconstructions, complications were very common at medium- to long-term follow ups. Thus, especially in reflection of a high postoperative prosthesis morbidity with potentially devastating complications, patients need to be carefully selected, thoroughly educated and integrated into the decision-making process (Figure 5).

Deep infection was the most prevalent complication after implantation, with 9 out of 20 patients (45%) experiencing this complication type, followed by aseptic prosthesis loosening in 4 out of 20 patients (20%) and dislocations with need of surgical revision in 3 out of 20 patients (15%). Thromboembolic events were particularly threatening and required immediate surgical revision in 3 out of 20 patients (15%). These results are similar to those of other studies describing outcomes after implantation of extensive custom-made prostheses, with an especially high prevalence of deep infections and aseptic loosening described in literature [21,22,23]. Although complications were frequent, literature shows acceptable but developable implant survival times of pelvic custom-made endoprostheses. Witte et al. presented a 3-year implant survival rate of 61.4%, while Holzapfel et al. showed an implant survival of 77% at five years [24,25]. At our institution, these high complication numbers led to a diminished use of custom-made endoprostheses at the expense of saddle endoprostheses or ice-cone shaped endoprostheses in the last decades [26,27]. These types of implants come with the inherent advantages of immediate availability, in comparison to a mandatory planning period for custom-made prostheses. However, not all types of periacetabular tumors may be addressed with saddle- or ice-cone shaped endoprostheses, as enough iliac bone is required for implant fixation. 

**Figure 6:**
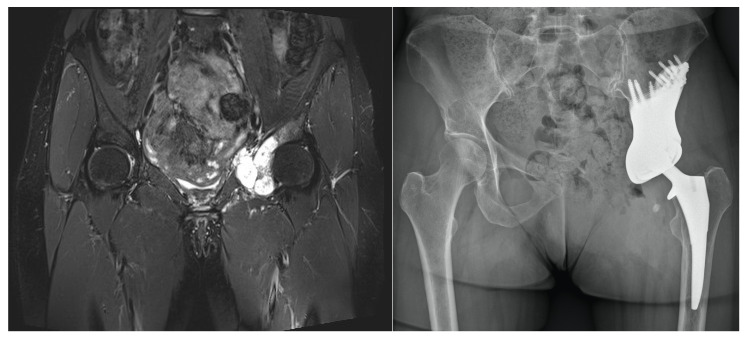
The patient suffered from chondrosarcoma (**left**) and received type II and III wide resection and implantation of a 3D-printed custom-made prosthesis with an Avantage cup (Zimmer Biomet, Warsaw, IN, USA) and Actis femoral stem (DePuy Synthes, Raynham, MA, USA) in August 2020. One year after surgery, the 50-year-old patient can walk for an hour with crutches, while smaller distances can be completed without walking aid. (**right**).

**Figure 7:**
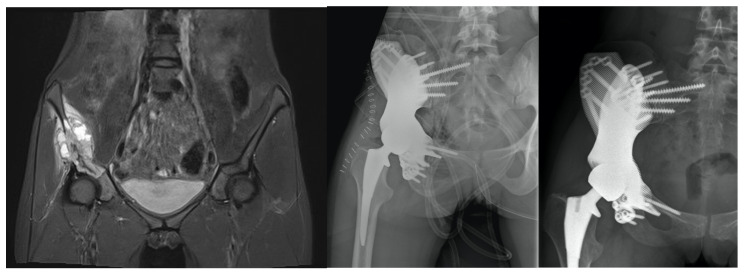
The patient suffered from an osteosarcoma (**left**) and received a type I and II wide resection and implantation of a 3D-printed Materialise custom-made prosthesis (Materialise, Gilching, GER). In this patient, a cemented Durasul inlay (Zimmer Biomet, Warsaw, IN, USA) articulates with an Actis femoral stem (DePuy Synthes, Raynham, MA, USA). (**middle**) Wound dehiscence led to surgical debridement four months after prosthesis implantation. Two years after surgery, the 24-year-old patient is pain free and has a moderate limb walk with no walking aid required. (**right**).

We believe that emerging 3D-printed custom-made prostheses show great promise in reconstruction of extensive periacetabular tumors due to a potential reduction of duration of surgery and thus postoperative complications, a higher prosthesis survival and stability and better availability due to a fast 3D-printing process (Figure 6 and Figure 7) [2,28]. Current, early studies show favorable results of 3D-printed implants, as Wang et al. reported no deep infection in 13 patients and Jovicic et al. showed no deep infections in 11 patients [2,29]. Additionally, modern concepts of osseointegration, such as osteoconductive, alveolar structures as well as improved porous surfaces between the implant-bone interface may easily be implemented in 3D-printed implants [8]. Another important aspect of 3D-printed implants is an advancement in dead space management, as 3D-printed design structures may lead to an improved soft tissue attachment and thus less soft tissue pouches.

### 4.3. Overall Oncological Survival after Resection of Extensive Pelvic Tumors

In relation to the necessary extend of resection and reconstruction required for wide resection margins and the median tumor size before resection, overall survival was acceptable with rates of 77% after one year, 69% after two years and 46% after five years. Although oncological survival comparisons after endoprosthetic pelvic reconstructions need to be cautiously evaluated due to high heterogeneity of underlying tumor entities and usually small patient numbers in other studies, these studies’ numbers went according with literature, as Wilson et al. reported a pooled mean 5-year patient survival of 55% (37.5–72%) in a recent systematic review [3]. Due to these numbers and a possibly high follow up period, we think that indications for custom-made reconstruction may be found especially in young and otherwise fit patients, due to functional demands and a higher probability to survive extensive pelvic surgeries.

### 4.4. Functional Results of Patients Mobilized with Custom-Made Prostheses

In frontiers of limb salvage surgery, functional outcomes are of particular importance to justify invasive and complication-ridden procedures. In this context, this study showed good functional results, with a median HHS of 81 (37–92) points at time of last follow up. More than half of all patients showed a high weight bearing capability, as six patients walked without walking aid and six other patients only needed one walking stick. Reports of acceptable to good functional results after extensive pelvic reconstruction are common in the literature. In a retrospective case series, Abudu et al. reported a mean MSTS-93 score of 21 out of 30 points in 35 patients, while Jaiswal et al. showed a mean TESS of 59.4% in 98 patients [22,30]. These results underline the need of stable pelvic constructs, as functional outcomes are good and desirable in oncologic patients surviving their disease. 

## 5. Conclusions

Internal hemipelvectomy and reconstruction using custom-made implants comes with a high risk for postoperative complications. However, good functional outcomes can be regularly achieved. This information may help treating surgeons to find adequate indications, as eligible patients need to be critically selected. Future studies evaluating new generations of 3D-printed custom-made pelvic implants are needed to determine their clinical value.

## Figures and Tables

**Table 1:** Demographic data.

Parameter	Patients (*n* = 20)	RFS
Median age at surgery	25 (13–63) years	0.9 ^T^
Median follow up after surgery	5 (1–17) years	0.7 ^T^
Sex		
Male/Female	9/11	0.7 *
Primary tumor size and localization		
Median tumor size	343 (min = 22, max = 3600) cm^3^	0.9 ^T^
Ilium	19	0.3 *
Pubis	1	
Tumor entity		
Chondrosarcoma	8	0.5 *
Ewing sarcoma	5	0.7 *
Osteosarcoma	4	0.6 *
PNET	2	0.2 *
Hemangiopericytoma	1	
Grade		
Low (G1–G2)	5	0.5 *
High (G3–G4)	14	0.3 *
N/A	1	

RFS = Revision free survival. ^T^ = T-test, * = Log-rank test

**Table 2:** Demographic statistics of patients included and lost to follow up.

Parameter	Included (*n* = 20)	Lost to Follow Up (*n* = 6)	*p*
Median Age at surgery	25 (13–63) years	40 (10-61) years	0.7 ^T^
Follow up after surgery	5 (1–17) years	3 (0-8) months	0.007 ^T^
Sex			
Male/Female	9/11	2/4	0.6 ^#^
Oncological status			
No evidence of disease	12 (60%)	0 (0%)	0.01 ^#^
Dead of disease	7 (35%)	6 (100%)	0.005 ^#^
Dead of other cause	1 (5%)	0 (0%)	
Conversion to hemipelvectomy	3 (15%)	3 (50%)	0.07 ^#^
Infection	9 (45%)	3 (50%)	0.8 ^#^
Thromboembolic event	5 (25%)	2 (33%)	0.7 ^#^
Conservative/surgical treatment	2/3	0/2	

Differences between groups tested via, ^T^ = T-test, ^#^ = Chi-square-test.

**Table 3:** Complications leading to surgical revisions.

Surgical Complications	Patients (*n* = 20)	Median Time to Revision
Median sum surgical revisions per patient	1.5 (0–7)	
Prosthesis explantation	3	15 months (95 days–16 years)
Deep infection	9	86 days (13 days–5 years)
Aseptic loosening	4	38.5 (10–80) months
Dislocation	3	5 months (14 days–20 months)
Sciatic nerve lesions	4	26 (24–28) months
Conservative/surgical treatment	2/2	
Thromboembolic event	5	0 (0–0) days
Conservative/surgical treatment	2/3	

**Table 4:** Surgical parameters.

Parameter	Patients (*n* = 20)	RFS
Surgical approach		
Ventral + Dorsal	12	0.9 *
Ventral	7	0.5 *
N/A	1	
Type of internal hemipelvectomy (Enneking/Dunham)		
I–IV	8	0.6 *
I, II, III	3	0.6 *
I, II	3	0.5 *
II, III	2	0.8 *
I, II, IV	2	0.7 *
N/A	2	0.5 *
Femoral stem		
Zweymueller	12	0.7 *
Austroprosthesis	3	0.5 *
N/A	5	0.9 *
Cemented Polyethylene Cup		
Brunswick	9	0.4 *
N/A	9	0.9 *
Mueller	2	0.3 *
Surgical margin		
Negative	19	0.6 *
Positive	1	
Oncologic status at final follow up		
No evidence of disease	12	0.7 *
Dead of disease	7	0.3 *
Dead of other cause	1	
Functional status at final follow up		
Median Harris Hip Score (*n* = 11)	81 (37–92) points	
Mobilized with hip orthosis	4	0.2 *
Walking without aid	6	0.4 *
Walking with a walking stick	6	0.98 *
Walking with two crutches	3	0.1 *
No information	5	0.93 *

RFS = Revision free survival. * = Log-rank test

## Data Availability

The datasets used for this study are available from the corresponding author on reasonable request.

## References

[1] Pant R., Moreau P., Ilyas I., Paramasivan O.N., Younge D. (2001). Pelvic limb-salvage surgery for malignant tumors. Int. Orthop..

[2] Wang J., Min L., Lu M., Zhang Y., Wang Y., Luo Y., Zhou Y., Duan H., Tu C. (2020). What are the Complications of Three-dimensionally Printed, Custom-made, Integrative Hemipelvic Endoprostheses in Patients with Primary Malignancies Involving the Acetabulum, and What is the Function of These Patients?. Clin. Orthop. Relat. Res..

[3] Wilson R.J., Freeman T.H., Halpern J.L., Schwartz H.S., Holt G.E. (2018). Surgical Outcomes After Limb-Sparing Resection and Reconstruction for Pelvic Sarcoma: A Systematic Review. JBJS Rev..

[4] Enneking W.F., Dunham W.K. (1978). Resection and reconstruction for primary neoplasms involving the innominate bone. J. Bone Jt. Surg. Am..

[5] Campanacci D., Chacon S., Mondanelli N., Beltrami G., Scoccianti G., Caff G., Frenos F., Capanna R. (2012). Pelvic massive allograft reconstruction after bone tumour resection. Int. Orthop..

[6] Gebert C., Wessling M., Hoffmann C., Roedl R., Winkelmann W., Gosheger G., Hardes J. (2011). Hip transposition as a limb salvage procedure following the resection of periacetabular tumors. J. Surg. Oncol..

[7] Donati D., Di Bella C., Frisoni T., Cevolani L., DeGroot H. (2011). Alloprosthetic composite is a suitable reconstruction after periacetabular tumor resection. Clin. Orthop. Relat. Res..

[8] Wang J., Min L., Lu M., Zhang Y., Wang Y., Luo Y., Zhou Y., Duan H., Tu C. (2019). Three-dimensional-printed custom-made hemipelvic endoprosthesis for primary malignancies involving acetabulum: The design solution and surgical techniques. J. Orthop. Surg. Res..

[9] Donati D., D’Apote G., Boschi M., Cevolani L., Benedetti M.G. (2012). Clinical and functional outcomes of the saddle prosthesis. J. Orthop. Traumatol..

[10] Fisher N.E., Patton J.T., Grimer R.J., Porter D., Jeys L., Tillman R.M., Abudu A., Carter S.R. (2011). Ice-cream cone reconstruction of the pelvis: A new type of pelvic replacement: Early results. J. Bone Jt. Surg. Br..

[11] Windhager R., Karner J., Kutschera H.P., Polterauer P., Salzer-Kuntschik M., Kotz R. (1996). Limb salvage in periacetabular sarcomas: Review of 21 consecutive cases. Clin. Orthop. Relat. Res..

[12] Khan F.A., Lipman J.D., Pearle A.D., Boland P.J., Healey J.H. (2013). Surgical technique: Computer-generated custom jigs improve accuracy of wide resection of bone tumors. Clin. Orthop. Relat. Res..

[13] Windhager R., Leithner A., Hochegger M. (2006). Revision of tumour endoprostheses around the knee joint. Review and own results. Orthopade.

[14] Puchner S.E., Funovics P.T., Böhler C., Kaider A., Stihsen C., Hobusch G.M., Panotopoulos J., Windhager R. (2017). Oncological and surgical outcome after treatment of pelvic sarcomas. PLoS ONE.

[15] Henderson E.R., O’Connor M.I., Ruggieri P., Windhager R., Funovics P.T., Gibbons C.L., Guo W., Hornicek F.J., Temple H.T., Letson G.D. (2014). Classification of failure of limb salvage after reconstructive surgery for bone tumours: A modified system Including biological and expandable reconstructions. Bone Jt. J..

[16] Harris W.H. (1969). Traumatic arthritis of the hip after dislocation and acetabular fractures: Treatment by mold arthroplasty. An end-result study using a new method of result evaluation. J. Bone Jt. Surg. Am..

[17] Chua A.W., Chua M.J., Kam P.C., Broekhuis D., Karunaratne S., Stalley P.D. (2019). Anaesthetic challenges for pelvic reconstruction with custom three-dimensional-printed titanium implants: A retrospective cohort study. Anaesth. Intensive Care.

[18] Fröschen F.S., Randau T.M., Hischebeth G.T.R., Gravius N., Gravius S., Walter S.G. (2020). Mid-term results after revision total hip arthroplasty with custom-made acetabular implants in patients with Paprosky III acetabular bone loss. Arch. Orthop. Trauma Surg..

[19] Burastero G., Cavagnaro L., Chiarlone F., Zanirato A., Mosconi L., Felli L., de Lorenzo F.D.R. (2020). Clinical study of outcomes after revision surgery using porous titanium custom-made implants for severe acetabular septic bone defects. Int. Orthop..

[20] Chiarlone F., Zanirato A., Cavagnaro L., Alessio-Mazzola M., Felli L., Burastero G. (2020). Acetabular custom-made implants for severe acetabular bone defect in revision total hip arthroplasty: A systematic review of the literature. Arch. Orthop. Trauma Surg..

[21] Dai K.R., Yan M.N., Zhu Z.A., Sun Y.H. (2007). Computer-aided custom-made hemipelvic prosthesis used in extensive pelvic lesions. J. Arthroplast..

[22] Abudu A., Grimer R.J., Cannon S.R., Carter S.R., Sneath R.S. (1997). Reconstruction of the hemipelvis after the excision of malignant tumours. Complications and functional outcome of prostheses. J. Bone Jt. Surg. Br..

[23] Ozaki T., Hoffmann C., Hillmann A., Gosheger G., Lindner N., Winkelmann W. (2002). Implantation of hemipelvic prosthesis after resection of sarcoma. Clin. Orthop. Relat. Res..

[24] Holzapfel B.M., Pilge H., Prodinger P.M., Toepfer A., Mayer-Wagner S., Hutmacher D.W., von Eisenhart-Rothe R., Rudert M., Gradinger R., Rechl H. (2014). Customised osteotomy guides and endoprosthetic reconstruction for periacetabular tumours. Int. Orthop..

[25] Witte D., Bernd L., Bruns J., Gosheger G., Hardes J., Hartwig E., Lehner B., Melcher I., Mutschler W., Schulte M. (2009). Limb-salvage reconstruction with MUTARS hemipelvic endoprosthesis: A prospective multicenter study. Eur. J. Surg. Oncol..

[26] Barrientos-Ruiz I., Ortiz-Cruz E.J., Peleteiro-Pensado M. (2017). Reconstruction After Hemipelvectomy With the Ice-Cream Cone Prosthesis: What Are the Short-term Clinical Results?. Clin. Orthop. Relat. Res..

[27] Jansen J.A., van de Sande M.A.J., Dijkstra P.D.S. (2013). Poor long-term clinical results of saddle prosthesis after resection of periacetabular tumors. Clin. Orthop. Relat. Res..

[28] Wang B., Hao Y., Pu F., Jiang W., Shao Z. (2018). Computer-aided designed, three dimensional-printed hemipelvic prosthesis for peri-acetabular malignant bone tumour. Int. Orthop..

[29] Jovičić M.S., Vuletić F., Ribičić T., Šimunić S., Petrović T., Kolundžić R. (2021). Implementation of the three-dimensional printing technology in treatment of bone tumours: A case series. Int. Orthop..

[30] Jaiswal P.K., Aston W.J., Grimer R.J., Abudu A., Carter S., Blunn G., Briggs T.W., Cannon S. (2008). Peri-acetabular resection and endoprosthetic reconstruction for tumours of the acetabulum. J. Bone Jt. Surg. Br..

